# Maladaptive cognitions and physical health of the caregivers of dementia: An interpretative phenomenological analysis

**DOI:** 10.3402/qhw.v10.28980

**Published:** 2015-09-16

**Authors:** Sidra Ali, Iram Z. Bokharey

**Affiliations:** 1 Centre for Clinical Psychology, University of the Punjab, Lahore, Pakistan; 2 Services Hospital Lahore, Lahore, Pakistan

**Keywords:** Caregivers, dementia, qualitative research

## Abstract

The aim of the study was to conduct in-depth analyses of the lived experiences of the caregivers of dementia and their maladaptive thinking patterns and how their physical health was influenced and compromised. The main method used was interpretative phenomenological analysis and involved in-depth analysis of eight participants screened through homogenous purposive sampling. After taking written consent from the participants, semi-structured interviews were conducted to gather the data that were transcribed later on to carry out free textual analysis. The themes were generated from the transcripts through the funneling approach in order to arrive at the themes that were common, frequent, and reflected the experiences shared by the participants. The verification was done through peer review and rich thick description. The most significant themes regarding maladaptive cognitions were catastrophizing, overgeneralizing, and blaming, whereas fatigue and sleep disturbances were the most significant themes regarding physical health. The emergent themes point towards a need to devise indigenous therapeutic intervention for the caregivers of dementia in the Pakistani sociocultural context as the literature available on caregiving is quite scanty in our culture.

Aging is a natural phenomenon, and most of us may have an older person in our families suffering from serious chronic physical or cognitive impairment. The rise of dementia in South Asian countries poses a challenge in view of the poor infrastructure of the health care system in these countries as well as lack of insight about such diseases among general masses (Qadir, Gulzar, Haqqani, & Khalid, 2013). Pakistan was ranked 20th by the World Health Organization data for expiries due to dementia (World Life Expectancy, 2012). Family is a strong institution in Pakistan, and consequently, it is expected to take care of the elderly at home. Most people live in a joint family system where the older adults are highly honored by all family members on account of religious and cultural values (Shaji, George, Prince, & Jacob, 2009; Sulman, 2008).

In Pakistan, the family as an institution of caregiving is valued to be the backbone of the health care system as it serves to provide care to the patient by family members such as parents, spouses, or close relatives (Imran et al., 2010). The expeditious economic and social turnover tend to exert strain on the family system (Patel & Prince, 2001; Shaji, 2009; Shaji et al., 2009) and, consequently, lead to global changes in the family structure and size by redefining the roles of the members (Jathanna, Latha, & Bhandary, 2010). Providing care to someone with chronic disability is traditionally gendered in women in Asian Indian families (Rozario, Morrow-Howell, & Hinterlong, 2004). According to feminist approach, the contributions of women in India in providing care for the elderly are marginalized both at sociopolitical and familial level, as the role of men is domineering in the society (Beasley, 1999). The rapidly increasing drastic changes in the roles and value system, urbanization, or migration for better resources are major reasons to cause erosion of traditional family system support and this demographic shift will devastate the values for older adults’ caregiving by leaving them uncared for (Patel & Prince, 2001).

Moreover, the potential to fulfill the demands of the caregiving situation within the available limited resources tends to be correlated with the quality of life of both the caregiver and care-recipient, as dealing with the burden might be reflected through cultural and social norms (De Vugt et al., 2005). It seems that the impact of caregiving on the caregivers depends on how they maintain a balance between caregiving activities and personal tasks. At times, caregivers contribute their time and energy at the cost of their own well-being. This negatively impacts their social activities and quality of life, and creates burden, physical health–related issues, and psychological morbidity (Shah, Wadoo, & Latoo, 2010).

The information gathered from the literature also indicates that the caregivers of dementia have significantly low self-efficacy, poor physical health, and subjective well-being as compared to other caregivers. Several factors that might contribute to psychological morbidity might include environmental stressors, physical and psychiatric history of illness, poor self-esteem, and severity of psychosocial and behavioral problems of patients with dementia (Brodaty & Donkin, 2009). Providing care can be so distressing and burdensome for the family members that they display high incidence of psychiatric morbidity such as anxiety and depressive disorders (Schulz & Martire, 2004). It was reported that the caregivers of dementia experience greater strain and stress, higher level of emotional burden, serious physical and mental health problems, and interpersonal conflicts (Cassie & Sanders, 2008). Another chronic issue faced by the caregivers tends to be disruption in sleep, especially among women. Moreover, it was found among 60% of women caregivers that the frequency of disruption of sleep was at least thrice a week (Smale & Dupuis, 2004). The strenuous process of providing care may interfere with nutrition, and have negative impact on the caregivers’ health, leading to physical ailments such as hypertension and respiratory issues, or may trigger excessive smoking or drinking, lack of exercise, and impaired immune functioning (Graesel, 2002).

The maladaptive ways of processing information is one of the major reasons contributing to a negative psychological state in terms of generating unrealistic goals, and maladaptive emotions and behaviors (Leahy, 2003). The dysfunctional thoughts and the maladaptive schemas built on account of the caregiving experience might have a negative impact on adaptability, adjustability, and well-being of the caregivers (Losada, Montorio, Knight, Marquez, & Izal, 2006). It is also evident from literature that dysfunctional thoughts of the caregivers regarding caregiving, both specific (Losada, Montorio, Izal, & Marquez, 2006 as cited in Rodriguez-Sanchez et al., 2012) or unspecific (Stebbins & Pakenham, 2001) are significantly related with distress.

Perhaps a large number of people with dementia remain undiagnosed, as in our culture patients are usually taken care of by close family members (informal caregivers) at home, no matter how serious the illness is, in order to avoid stigmatization (Wilkinson & Bowes, 2003). The rationale to conduct the study was to derive common themes shared by the caregivers in providing care to the patients in our particular social and cultural context. Therefore, the study aimed to explore the lived experiences of the caregivers that tend to have negative impact on their cognition, and its impact on their physical health.

## Method

The qualitative inquiry seemed best suited for this research as this was an exploratory study of a sensitive and rather complex phenomenon of caregiving. Initially, there was a need to carry out in-depth analyses on fewer participants of the significant aspects embedded in this important yet neglected area, which could only be addressed through qualitative approach. Later, these findings could form a base for quantitative studies on larger samples.

Last, the queries regarding maladaptive cognitions and physical health of the caregivers needed to be explored in a comprehensive manner, which was also one of the reasons why this study was situated in the qualitative paradigm.

### Interpretative phenomenological analysis

The strategy used in this qualitative inquiry was interpretative phenomenological analysis as it aims to explore meanings of particular experiences for the participants, that is, what sense participants make out of their social and personal world. It is a dynamic process that involves double hermeneutic, that is, combination of an empathic and questioning hermeneutic (Smith, 2008). According to Conrad (1987), the insider's perspective is explored that doesn't seem possible directly (as cited in Smith, 2008).

### Participants/sampling

Homogeneous purposive sampling was used as it involves selecting specific people with similar characteristics. It is a widely used sampling strategy in qualitative research to identify and select enriched cases about a particular phenomenon of interest (Palinkas, Horwitz, Green, Wisdom, & Hoagwood, 2015). We chose eight participants that actually proved to be a challenging and painstaking task, taking 3 months to trace the first participant. Adult informal caregivers who were actively engaged in caregiving for at least 6 months were referred by the psychiatrist from a hospital of Lahore, Pakistan.

### Procedure

The data were collected through in-depth semi-structured interviews as it allowed the researchers to conduct intensive individual interviews through open-ended questions to delve deeply into the participants’ caregiving experiences and to elicit clear pictures from participants’ perspectives (Boyce & Neale, 2006; DiCicco-Bloom & Crabtree, 2006; Guion, Diehl, & McDonald, 2011). The research guide constituted both general and specific questions to ease the flow of communication. To elicit rich emerging themes, the specific open-ended questions were put forward to make the interview iterative and to get satisfactory responses from the participants. The interviews were recorded to avoid missing significant details about the participants’ experiences, and were transcribed later on.

The transcripts were read in detail a couple of times in order to get familiarity with the account that was close to being a free textual analysis. Then a two-column table was formed by dividing the content into meaningful units and assigning a comment for each unit; the other margin was used to document the emerging theme titles. The initial notes were transformed into concise phrases which aimed to capture the essential quality that was found in the text. The themes helped to move the response to a high level of abstraction and invoke psychological terminology (Smith, 2008). The emergent themes were listed separately and then we tried to make connections between them and made clusters of some of the themes. Later a table of the themes was produced in an order and the clusters were themselves given a name and represented the superordinate themes. The themes were generated from the transcripts through the funneling approach, that is, moving from broader themes to narrow ones (Smith, 2008).

### Data analyses and verification

In order to analyze the lived experiences, their cognitions, and impact on physical health of the caregivers, an in-depth analysis was done. We went through the transcripts several times to explore the underlying meanings. Later we developed and transformed emergent themes and their interconnections were established and clusters formed (Smith, 2008). To enhance the authenticity and credibility of the research the data were verified through peer review and rich thick description (Creswell, 1998).

### Ethical considerations

This project was approved by the Departmental Doctoral Programme Committee at the Centre for Clinical Psychology, University of the Punjab, Lahore, Pakistan. Written informed consent was taken after ensuring confidentiality. The participants were briefed about the nature of the study and were given the right to withdraw at any point. They were informed that fictitious names would be assigned to them in the write up of the study. Moreover, they were also told that no monetary compensation would be given. Last, they were informed that if they experienced psychological distress during the interviews, free counseling services would be offered.

## Results and discussion

Table I and II summarize the basic information about the participants.

**Table I:** Demographic information regarding caregivers of patients of dementia

Name	Age (years)	Gender	Education	Marital status	Occupation	Total family members	Duration of caregiving
Shehla	51	Female	MA	Married	Housewife	5	6 years
Shahbaz	69	Male	ACCA	Married	Retired	6	1 year
Nazia	62	Female	MA	Married	Housewife	5	2 years
Saima	58	Female	BA	Married	Housewife	4	4 years
Shahid	64	Male	MBA	Married	Retired	6	3 years
Waqas	59	Male	MSc	Married	Retired	5	5 years
Omar	63	Male	MBA	Married	Retired	4	3 years
Aslam	56	Male	BSc	Married	Retired	6	4 years

**Table II:** Subthemes of maladaptive cognitions and physical health of caregivers

Title	Main theme	Subtheme
Cognitive distortions	Catastrophizing	Self
		Other
		Future
	Blaming	Self
		Family
		World
	Overgeneralizing	Personal domain
		Marital domain
		Social domain
		Familial domain
Physical health	Fatigue	Reduced physical fitness
		Locus of control directed towards powerful others
		Sense of loss of control
		Loss of time
		Overburdened schedule
	Sleep disturbance	Sleep pattern variability
		Effect of sleep on daytime functioning
		Attempts at coping and sleep management
		Frequent awakenings
		Bodily pain
		Mental alertness

### Theme I: Cognitive distortions

Cognitive distortion refers to errors in an individual's way of reasoning that may lead to faulty assumptions and misconceptions (Corey, 2009). The cognitive distortions are maladaptive thinking patterns displayed by an individual that has inadequate ground for being true. In the current study, they seemed to be responsible for generating negative unhealthy emotions and maladaptive behaviors and were a result of the excessive demands of the caregiving phenomenon. The participants found the process of caregiving laborious, troublesome, and annoying as it incapacitated their functioning because they were unable to achieve satisfaction from different domains (personal, marital, social, and familial) of life.

#### Catastrophizing

In this cognitive distortion, the person believes what has happened or will happen in the future would be so awful and unbearable that he/she won't be able to stand it (Leahy, 2003). In making the error of catastrophizing, the participants actually believed that a negativity would occur in the future and would be disastrous or unendurable for them. This faulty thinking ultimately led them to experience unhealthy negative emotions like frustration, fears, and apprehensions. Nazia reported having apprehensions regarding her mother's health and illness. She felt frustrated and overwhelmed around her mother's actions because her mother used to keep her alert all day long. Nazia shared:

The caregiving experience was a traumatic, horrible and terrible experience of my life which had a negative impact on my physical health as I had disturbed patterns of sleep. I perceived myself incapable to stand the circumstances. Whenever my mother left her room I was unable to keep an eye on her all the time, which was quite traumatic and distressing for me as I was scared that she may go near the heater/stove and may catch fire or she may go somewhere else and get into trouble or she may do anything wrong with herself that may prove to be an unbearable loss. Oh God! I can't even get sound sleep due to these apprehensions.

Similarly, Omar reported to be quite worried and had fear of loss regarding his wife. In order to provide caregiving he used to be with her all day and night. Omar's wife used to place cooked food at improper places such as in cabinets and cupboards, left the stove burner on, and consequently, often burned meals. For instance, he shared:

She often misplaced money at home or lost it in the market. I always anticipated something terrible happening to my wife or at home especially when I was away which would be unbearable or traumatic for me. My mind is obsessed that something is going wrong and it will continue to occur with the passage of time because she (wife) was not able to keep things at proper places. I got fearful that God forbid something worst might not happen as I am totally dependent upon her. I am a one woman man so in that case I often think of the worst. So it's a matter of great fear.

#### Blaming

Blaming was another cognitive distortion apparent in the transcripts. The individual displaying this focuses on the other person as the source of his negative feelings and refuses to take full responsibility for changing himself/herself such as “she's to blame for the way I feel now” or “my family is responsible for all my problems.” The individual usually keeps track of blaming self or others for every problems (Leahy, 2003). It seemed that instead of being mindful for thinking and feeling in a negative way, the participants attributed the blame to other family members and the care-recipient for being responsible for the unpleasant feelings, constraints, and distress that they were facing. However, at the same time they were also taking the responsibility of caregiving.

For example, Saima stated that she had to compromise a lot as caregiving was imposed on her by her husband. She often criticized others for her problems and assumed that others were responsible for her miserable condition; she often blamed her brother-in-law for the care-recipient's illness. Her brother-in-law also shared the burden with Saima during her critical time but he became inaccessible shortly afterwards. It seems that Saima had a strong need for succorance as she perceived her physical and psychological health to be at stake. She said:

I used to blame my husband as I had to face the crisis due to my husband's unilateral decisions for which I had to face the negative consequences all along my life. I was even deprived of marital satisfaction. Besides, no one was willing to help in the process of caregiving. I often said to my husband that he didn't care about me. He didn't think about me. He was not concerned about me. I don't have my personal life. I often blame him and even myself.

Nazia shared that she often had arguments with her mother as she perceived her mother to be the root cause of all problems in her life. Her mother was reported to be arrogant, selfish, self-centered, and exhibited extreme self-love. Her mother had been the authority figure and usually made unilateral decisions which suited her only. Nazia shared that her mother used to devalue and criticize her even in the presence of relatives and guests and didn't miss a chance to humiliate and discourage her if she didn't perform some task appropriately. Nazia's mother used to discriminate between her grandsons also which really disturbed her younger son. Ultimately, her younger son became so distressed that he left home and refused to get married as he had negative views about women due to his poor interaction with grandmother. It seemed that Nazia analyzed the situation without removing her biases; rather she disavowed her responsibility in all the circumstances. As she shared:

When I analyzed the circumstances, I came up with the conclusion that all the hardships I have to face in my personal, social, and marital life were due to my mother. I often blame my mother and often have argumentation with her that I spent a tough time due to her and I even couldn't go to meet my son who was living abroad. I have restricted socialization as I was not willing to take her (mother) with me but she (mother) insisted to attend social gatherings that usually proved to be an embarrassing situation for me.

#### Overgeneralizing

Overgeneralizing means that an individual perceives a global pattern of negatives on the basis of a single incident as a never-ending pattern of defeat. For instance, I seem to fail at a lot of things (Leahy, 2003). If something bad or worse happens with someone only once, it is generally expected to happen over and over again. It was evident from the data that the participants perceived the process of caregiving as a never-ending pattern of worst consequences that had a huge negative impact on the rest of the domains that were fully functioning before the patient's illness. Waqas reported that the deterioration in the care-recipient's functioning made me more worried when she started losing money, mismanaging the household chores such as burning of meal, left the burner on, and misplacing things rather frequently. His children didn't show any concern and refused to share the responsibility. His daughter was supposed to help the mother in the kitchen, but she had a very careless and dispassionate attitude in this regard. The whole scenario caused a great disturbance in home management. His pension was not enough to meet the demands of caregiving and he was psychologically distressed over the situation and felt frustrated over the negligence of his children. Waqas shared that:

I was worried upon the damages done on almost daily basis and it was getting worse with each passing day. I individually tried my best to overcome the situation but to no avail, I could not convince my family members at all to share the responsibilities. I was extremely distressed over what was happening to me. I was all the time preoccupied that things were getting worse. I was worried all the time about my wife regarding her deterioration that she may go down further in the future.

Shehla reported that caregiving impacted all the facets and domains of her life in a negative way. She often used to ignore her needs so that she could better manage her mother-in-law. However, the demands of caregiving deteriorated Shehla's health. As she reported:

My enjoyment is over. I have suffered a lot due to mother-in-law, my children are also affected. I don't have my own life and have seen the consequences of sacrificing. If I don't do anything for my health and kids then things will go worse. I preferred the needs of mother-in-law over my children that also adversely affected their psychological health. I ultimately realized that if I continued practicing that maladaptive pattern, it would continue to happen all along.

The above mentioned findings are in line with the study conducted on Alzheimer's caregivers as it was found that the dysfunctional thoughts among the caregivers are associated with their poor health, external locus of control, and depression (McNaughton, Patterson, Smith, & Grant, 1995).

### Theme II: Physical symptoms

It is a subjective state that refers to the degree of impairment of normal physiological function affecting part or all of an organism either temporarily or chronically. It seemed that this kind of a disabled state might have had impact on cognitions and vice versa. The lived experiences of the participants depicted that maladaptive cognitions had an adverse impact on their health by making them vulnerable to physical symptoms such as fatigue and disturbed sleep. Another major cause for these physical symptoms might be lack of social support as all the participants were managing the caregiving alone.

#### Fatigue

Fatigue is generally a symptom and not a disease in itself. It is defined as a physical and/or mental state of lack of energy and motivation, weariness, listlessness, and malaise that can be physical, mental, or both, and makes it difficult to perform ordinary tasks (Davis, 2010). Fatigue had an adverse impact on physical and mental states of the participants due to low energy level to perform everyday tasks. For instance, Shahbaz reported feeling physically and mentally exhausted due to the prolonged and never ending phenomena of caregiving that ultimately led to physical symptoms. He had loss of interest in outdoor activities and low activity level. Shahbaz shared:

I tried to keep myself engaged in social and personal activities but couldn't keep my interest and easily got exhausted. I don't have stamina to do work anymore. I get tired quite often. I get mentally exhausted. I lose interest quite often. Whenever I want to do something, I can't do that. My life is not much active. I am not interested even in outing and shopping. I get exhausted quite soon and even if I have to do something I couldn't find energy to do that.

Saima reported being overloaded by the constant pressure of caregiving that contributed to make her physically vulnerable. She found difficulty in handling her mother especially when she had to move her somewhere. Due to constant pressure of imposed tasks, Saima's backache became worse because she readily suffered from physical fatigue. It seemed to her that there was no end to her pain and misery. She expressed her views as:

My mother was quite hypertensive. She was taking medicines to reduce hypertension which were sleep inducing. So she started drawling left or right when I had to take the mother to the washroom and she had a healthy physique. That was a painful process because she used to put all her weight upon me. I was already suffering from backache. That was quite a tough time. I was suffering from both physical and mental fatigue due to these circumstances.

Due to fatigue, the participants felt incapacitated to provide supervision and fulfill their own needs which induced distress in their lives.

#### Sleep disturbance

It is a symptom or condition that refers to lack of the necessary amount of sleep taken by the individual due to the individual's own body and mind, or by another individual (Peter, 2012). This condition interferes with normal sleep and causes difficulty in falling or staying asleep. The disturbed pattern of sleep may be either acute or chronic and may be due to physiological or psychological factors or fundamental aspects of sleep hygiene. If the sleep disturbance is induced by stress, the rate of sleep disturbance depends on the severity and duration of the stressful situation. It might be due to pressure of an uncongenial environment that the quality and rate of sleep of participants seemed to be inversely proportional to the distress in their environment. Aslam shared that his father had difficulty in falling asleep and used to interrupt him often by knocking at his door. It used to happen multiple times during most nights. Consequently, he couldn't find time to have an adequate amount of sleep. He often had to spend sleepless nights supervising his father and this pattern was reported to persist for a prolonged time period. Aslam said:

My father's illness often gave me sleepless nights. He used to constantly knock at my door at midnight after every few minutes and then started wandering in the home that was quite distressing and frustrating. I started remaining so worried that couldn't take proper sleep, sometimes for many days at a stretch. When I got up in the morning, I felt tired, frustrated, and irritable due to sleep deficiency that led to a disturbed routine for the entire day.

Shahid shared that he felt frustrated and had a low mood as his quantity and quality of sleep was compromised. He used to spend the major part of the day and night serving his wife. As his wife had difficulty falling asleep, Shahid had to stay awake until she fell asleep. Therefore, he could only sleep for a couple of hours at night, but had to get up early in the morning for the household chores. All this negatively impacted his physical health. He shared that:

The most serious issue for me is sleep disturbance that is increasing but I have to get up for my wife. I have to sit beside her till late at night until she falls asleep. I even get up in the middle of night to see whether my wife has gotten into any trouble or may need something. So I get up multiple times during sleep that's why unable to fulfill the requirement of sleep. I need to get up early in the morning to supervise my wife and carry out the household chores.

Prolonged disturbances in sleep patterns of participants impaired the natural phenomena of physical and psychological repair during the sleep state. This stage ultimately influenced the participants’ attitudes and behaviors towards self, world, and future and made them emotionally and psychologically vulnerable to illness. These findings are supported by other research in which it was found that the quality of life of the participants providing caregiving to the Alzheimer's patients was compromised, and the difficulties regarding quality of life, mental health, and family burden were noticeable (Imran et al., 2010; Kim, Zarit, Femia, & Savla, 2012).

We found that the disturbed sleep patterns among the participants and their physical health status contributed to caregiver burden as literature on sleep disturbances and their contributing factors among caregivers revealed that disturbed sleep routine, physical health status, burden, and depression among caregivers are interconnected and interdependent (McCurry, Logdson, Teri, & Vitiello, 2007).

From the above discussion, it seems that there was an interplay among maladaptive cognitions and physical symptoms of the participants, and that these psychosocial factors were directly proportional to the participants’ perceived level of burden and compromised quality of life, and had a negative impact on the global functioning of the participants.

There is empirical evidence that low self-perceived health, emotional support, and greater burden level among caregivers was associated with the high number of hours spent each day in caregiving. The poor mental health, chronic diseases, and physical symptoms of the caregivers were associated with more physical support provided to the patients (Chang, Chiou, & Chen, 2010). Besides that, men and women caregivers were reported to have low back pain and poor psychological health in comparison to men and women non-caregivers (Yiengprugsawan, Harley, Seubsman, & Sleigh, 2012). A meta-analysis revealed that the higher level of behavior problems exhibited by the care-recipient correlated with poor health of the caregiver. Moreover, the caregivers experiencing stressors related to dementia tend to have negative impact of caregiving on their physical health (Pinquart & Sorensen, 2007).

To have a mirror view regarding the adaptation of the participants in their environment and adjustment in significant domains of life, it is important to analyze the discrepancy between the demands of a role and their limited resources. Ecological Systems Theory (as cited in Wilder, 2009) postulates how a family or an individual fits in his environment. The theory emphasizes that if the individual connects and engages in a supportive environment, his functioning improves. In order to examine the best fit of an individual/family in an environment, there is a need to examine the amount of social support available to that person in the existing environment. With regard to the experiences of the participants in the present study, the demands of the caregiving seemed to be incompatible with the available resources because the stressors were perceived as a crisis which the participants had to manage alone.

The findings of our study correlated well with the salient features of Exchange Theory (as cited in Durant & Christian, 2006) as it postulates that the relationship between the caregiver and care-recipient depends on their capacity to reward something of value to each other that may be approval, affection, information, money, housework, or anything else. When the resources to be exchanged are compatible between the two persons, they will share mutually satisfying interdependence. However, if an individual has fewer resources to exchange, the ability to get profit from the other person will be restricted and the exchange relationship won't be balanced out and ultimately would lead to negative consequences. The participants gave time, energy, money, performed household tasks incessantly, limited their social life, ignored their personal needs, and compromised with physical ailments. On the other hand, the care-recipient had little resources to reciprocate or exchange and this imbalance led to negative consequences such as fear of loss, frustration, isolation, desperation, burden, fatigue, and disturbed sleep.

## Conclusion

Thus, it might be concluded that the themes identified in this study were quite significant as they helped ascertain the cognitive framework of the caregivers of dementia and its impact on their physical health in our particular sociocultural context. The findings would help devise indigenous therapeutic intervention based on cognitive behavior therapy to facilitate the caregivers to cope more adequately with their extremely demanding and taxing routine. Last, a limitation of this study was that we were unable to address gender differences and future research needs to look into this.
